# Chronic Psychosocial Stress Impairs Bone Homeostasis: A Study in the Social Isolation Reared Rat

**DOI:** 10.3389/fphar.2016.00152

**Published:** 2016-06-08

**Authors:** Stefania Schiavone, Maria G. Morgese, Emanuela Mhillaj, Maria Bove, Angelo De Giorgi, Francesco P. Cantatore, Claudia Camerino, Paolo Tucci, Nicola Maffulli, Vincenzo Cuomo, Luigia Trabace

**Affiliations:** ^1^Department of Experimental and Clinical Medicine, University of FoggiaFoggia, Italy; ^2^Department of Physiology and Pharmacology, “Sapienza” University of RomeRome, Italy; ^3^Dual Diagnosis Unit, Azienda Sanitaria Locale della Provincia di FoggiaFoggia, Italy; ^4^Department of Medical and Surgical Sciences, University of FoggiaFoggia, Italy; ^5^Department of Basic Medical Science, Neuroscience and Sense Organs, University of BariBari, Italy; ^6^Department of Musculoskeletal Disorders, School of Medicine and Surgery, University of SalernoSalerno, Italy; ^7^Centre for Sports and Exercise Medicine, Barts and The London School of Medicine and DentistryLondon, UK

**Keywords:** social rearing isolation, bone homeostasis, cathepsin K, sclerostin, CTX-I

## Abstract

Chronic psychosocial stress is a key player in the onset and aggravation of mental diseases, including psychosis. Although a strong association between this psychiatric condition and other medical co-morbidities has been recently demonstrated, few data on the link between psychosis and bone homeostasis are actually available. The aim of this study was to investigate whether chronic psychosocial stress induced by 4 or 7 weeks of social isolation in drug-naïve male Wistar rats could alter bone homeostasis in terms of bone thickness, mineral density and content, as well as markers of bone formation and resorption (sclerostin, cathepsin K, and CTX-I). We found that bone mineral density was increased in rats exposed to 7 weeks of social isolation, while no differences were detected in bone mineral content and area. Moreover, 7 weeks of social isolation lead to increase of femur thickness with respect to controls, suggesting the development of a hyperostosis condition. Isolated rats showed no changes in sclerostin levels, a marker of bone formation, compared to grouped animals. Conversely, bone resorption markers were significantly altered after 7 weeks of social isolation in terms of decrease in cathepsin K and increase of CTX-I. No alterations were found after 4 weeks of isolation rearing. Our observations suggest that chronic psychosocial stress might affect bone homeostasis, more likely independently from drug treatment. Thus, the social isolation model might help to identify possible new therapeutic targets to treat the burden of chronic psychosocial stress and to attempt alternative therapy choices.

## Introduction

Chronic psychosocial stress is an important risk factor in the onset and aggravation of mental disorders, including psychosis. Thus, evidences from both clinical trials and rodent models show that humans at strong risk for psychosis or animals with behavioral and neuropathological alterations, reminiscent to psychotic symptoms, experienced high levels of psychosocial stress (Wisely et al., 2010; Pruessner et al., 2011; Mhillaj et al., 2015; Schiavone et al., 2015).

Accumulating evidence suggests that psychosis is also associated to alterations in skeletal status compared to healthy population (Montejo, 2010; Nousen et al., 2013). However, bone metabolism dysregulation in psychotic patients (Halbreich, 2007; Lin et al., 2015) has only recently received attention. In this context, a decrease in bone mass is significantly more common in patients with schizophrenia compared to control subjects (Stubbs et al., 2014). Skeletal fragility has been associated to pharmacological treatment, in particular to antipsychotic drugs (O’Keane and Meaney, 2005; Kishimoto et al., 2012). Thus, low bone mineral density (BMD) values have been observed in medicated psychotic patients (Baastrup et al., 1980; Halbreich et al., 1995), and a drug-induced decrease in BMD has been attributed mostly to hyper-prolactinemia and its consequences (Meaney and O’keane, 2002). Several studies on the relationship between antipsychotic drugs and BMD reduction in psychotic patients produced controversial results. Indeed, while several reports evidenced a statistically significant association (O’Keane and Meaney, 2005; Meaney and O’keane, 2007), others did not describe this relationship (Abraham et al., 2003; Becker et al., 2003; Howes et al., 2005; Bergemann et al., 2008). It is not clear whether psychotic patients are predisposed to certain alterations or whether these abnormalities are mainly treatment side-effects. Drug-naïve patients suffering from first psychotic episode actually represent a good opportunity to investigate the possible link between medication and the development of skeletal disorders. Unfortunately, only few studies are available from the pre-antipsychotic period, and problems with diagnosis and methodological concerns make their interpretation difficult (Crews et al., 2013). In particular, some studies have included as “drug-free” patients who in reality had received medication for a short period of time. Actually, in some cases, participants with psychosis were allowed to receive anti-anxiety medication (Fernandez-Egea et al., 2009). Since experimental methods and confounding variables can be minimized in animal models, the present study was designed to assess the potential development and prevalence of bone status abnormalities in the post-weaning social isolation rat model of psychosis, which provides a non-pharmacological tool to induce neurobiological and behavioral changes in animals, reminiscent of what observed in psychotic subjects (Bakshi and Geyer, 1999; Lapiz et al., 2003; Fone and Porkess, 2008).

Here, we investigate whether and how bone homeostasis could be impaired in an animal model of chronic psychosocial stress. To this purpose, we compared the skeletal status in animals reared either in social isolation (4 and 7 weeks) or in social groups, by using dual-energy X-ray (DEXA) absorptiometry. A microscopy investigation was also performed to assess possible bone thickness variation. Finally, to evaluate the activity of bone remodeling process, biochemical markers of bone formation (serum sclerostin) and resorption (serum cathepsin K and CTX-I) were measured.

## Materials and Methods

### Animals

Adult male and female Wistar rats (Harlan, S. Pietro al Natisone) weighting 250–280 g were housed at constant room temperature (22 ± 1°C) and relative humidity (55 ± 5%) under a 12 h light/dark cycle (lights on from 7:00 AM to 7:00 PM) for at least 7 days before the experiments. Food and water were available *ad libitum*. Procedures involving animals and their care were conducted in conformity with the institutional guidelines of the Italian Ministry of Health (D.L. 26/2014), the Guide for the Care and Use of Laboratory Animals: Eight Edition, the Guide for the Care and Use of Mammals in Neuroscience and Behavioral Research (National Research Council, 2004), the Directive 2010/63/EU of the European Parliament and of the Council of September 22, 2010, on the protection of animals used for scientific purposes. The experimental protocol was approved by the Italian Ministry of Health. Approval number was not applicable. All procedures involving animals were conducted in accordance to ARRIVE guidelines. Animal welfare was monitored daily through the entire period of experimental procedures. No signs of distress were evidenced and all efforts were made to minimize the number of animals used and their suffering.

### Social Isolation Protocol

For the social isolation procedure, one male and two females were housed together for mating (Leng et al., 2004). The social isolation procedure was performed on 20 male rats. This sample size was established based on literature evidence about this animal model. At weaning (postnatal day 21), pups were separated from their mothers and reared either as isolated rats (ISO; one rat per cage) or in reared in group control rats (GRP; three to four rats per cage). To avoid a litter effect, each litter contributed only one subject to the GRP and one subject to the ISO. All animals were reared in Plexiglas cages (ISO: 40.0 cm × 27.0 cm × 20.0 cm; GRP: 59.0 cm × 38.5 cm × 20.0 cm). Animals were disturbed only for cleaning purposes, which consisted of changing the cage once a week for ISO and GRP. Both ISO and GRP rats were housed in the same room, so that ISO rats maintained a visual, auditory, and olfactory contact with the other animals. All experiments were conducted at the end of 4 and 7 weeks of isolation rearing. For *post-mortem* analyses, animals were deeply anesthetized with Equithesin (3.6 ml/kg; composition: 1.2 g sodium pentobarbital; 5.3 g chloral hydrate; 2.7 g MgSO4; 49.5 ml propylene glycol; 12.5 ml ethanol, 58 ml distilled water) and euthanized by decapitation, as previously described (Tucci et al., 2014).

### Blindness of the Study

Researchers performing analysis were blind with respect to the rearing conditions. Indeed, it was not possible to deduce from the labeling whether an animal was isolated or not. The social isolation procedure was performed in a dedicated part of the animal facility, not accessible to the investigators during the entire period of the social isolation protocol. The blinding of the data was maintained until the analysis was terminated.

### *In Vivo* Dual Energy X-ray Absorptiometry Analysis

A total of 20 animals (10 control and 10 isolated rats) were anesthetized by an intraperitoneal injection of Equithesin. The rationale for choice of these specific anesthetic and route of administration was established based on our experience and literature evidence. BMD (g/cm^2^), bone mineral content (BMC, g) and bone area (cm^2^) were measured in each animal by means of DEXA with a body scan densitometer (Hologic Dexa Bone Densitometer, Hologic Italia S.R.L., Rome, Italy). Before measurements, body calibration scans were performed with the Hologic phantom for small animals. Animals were positioned ventrally with the forelimbs away from the trunk to scan the whole body. The appropriate software program for small animals (DEXA; L & R Hip Software Ver. 11.1 for Windows) was used. DEXA scans were performed at post-weaning weeks 4 and 7. After the scan, three regions of interest (ROI) were marked, namely the right femur (R1), the T9–L5 vertebrae (R2) and the L1–L6 vertebrae (R3). All animal images were scanned and analyzed by the same operator.

After DEXA scanning, rats were euthanized by decapitation. Blood was collected into heparinized serum tubes. Serum samples were allowed to clot for 60 min followed by centrifugation at 4000 rpm for 10 min. Samples were stored at -80°C for later use. Then, the right femur was removed, cleaned, weighted, and stored in 4% formaldehyde at 4°C until further analysis.

### Bone Thickness Measurement

Bone thickness values were calculated on transverse femur sections (0,2 cm) of control (*n* = 5) and isolated rats (*n* = 6), as previously described (Pelletier et al., 2004; Hayami et al., 2006). Briefly, femur sections were obtained by using an electric cutter and then stained with diaminobenzidine (DAB), chromogen (DAB betazoid chromogen kit, Biocare medical Concord, CA, USA; 1 ml per sample) and H_2_O_2_ revealing solution (30 μl per sample). After two washes in PBS, the femur sections were mounted on a glass support and analyzed for bone thickness values by using the Nikon Upright Microscope Eclipse software.

### Measurement of Serum Biochemical Parameters

Sera from five control and five isolated rats were analyzed for sclerostin, CTX-I and cathepsin K using ELISA kits provided by Cloud-Clone Corporation (Houston, TX, USA). Assays were performed according to the manufacturer’s instructions. Each sample analysis was performed in duplicate to avoid inter-assay variations.

### Statistical Analysis

All statistical analyses were performed using Graph Pad^®^ 6.0 for Windows. Data were analyzed by Student’s *t*-test. After data conversion to natural logarithm, regression analyses were performed as previously reported (Prentice et al., 1994). Regression analyses were performed using Microsoft Office Excel 2013 version. Differences were considered significant only when *P*-values were less than 0.05.

## Results

### Body Weight

Rats were weighed weekly throughout the whole experiment. The initial and final weights are shown in **Table 1**. There was no difference in weight between grouped and isolated rats at any stage of the experimental period (4 weeks: *P* = 0.590 and *P* = 0.802 for initial and final weight, respectively; 7 weeks: *P* = 0.085 and *P* = 0.350 for initial and final weight, respectively). Thus, social isolation did not induce alterations of total body weight, when maintained at a standard chow diet.

**Table 1:** Effect of social isolation on rat body weight.

**A**		
	**Initial body weight**	**Body weight after 4 W**
GRP	45.09 ± 1.02	221.10 ± 8.22
ISO	45.85 ± 0.92	218.90 ± 4.33
**B**		
	**Initial body weight**	**Body weight after 7 W**
GRP	48.26 ± 1.34	350.90 ± 7.94
ISO	45.56 ± 0.71	341.20 ± 6.41

*Body weight of isolated (ISO) and grouped (GRP) rats at the beginning of the study (initial body weight) and after either 4 weeks of social isolation **(A)** or 7 weeks of social isolation **(B)**.*

All animals increased their weight during the experiment. The body weight gain of isolated rats during the 4 and 7 weeks’ experimental period was similar to that noted in the control groups. Though individual food intake in grouped rats was not measured, total food supply in isolated animals was similar to that in the grouped rats. All animals in each group survived throughout the experiment and they showed no obvious clinical signs of morbidity.

### Impact of Social Isolation on Bone Mass Parameters

**FIGURE 1:**
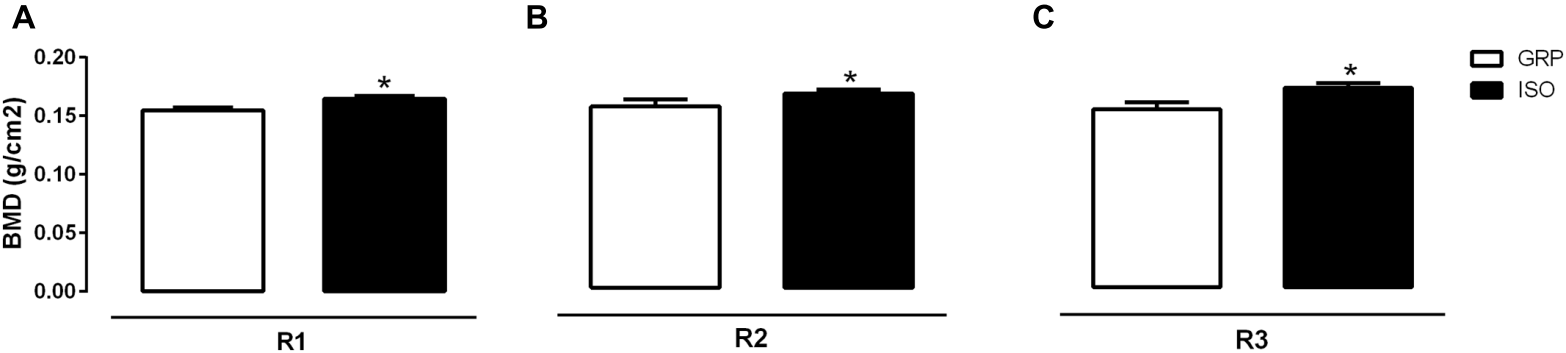
**Effect of social isolation on bone mineral density (BMD): Effect of 7 weeks of social isolation rearing on BMD (g/cm^2^) in region of interest femur (R1) **(A)**, T9–L5 vertebrae (R2) **(B)** and L1–L6 vertebrae R3 **(C)**; ^∗^*P* < 0.05, isolated (ISO) versus grouped (GRP) rats (*n* = 10 per group), unpaired student *t*-test**.

**FIGURE 2:**
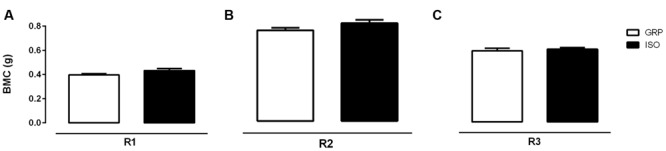
**Effect of social isolation on bone mineral content (BMC): Effect of 7 weeks of social isolation rearing on BMC (g) in region of interest femur (R1) **(A)**, T9–L5 vertebrae (R2) **(B)** and L1–L6 vertebrae R3 (C); *P* > 0.05, isolated (ISO) versus grouped (GRP) rats (*n* = 10 per group), unpaired student *t*-test**.

**FIGURE 3:**
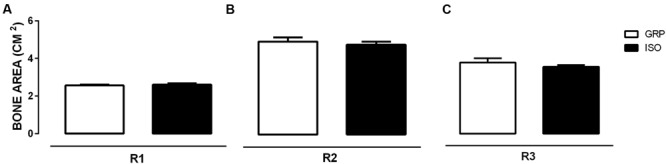
**Effect of social isolation on bone area: Effect of 7 weeks of social isolation rearing on bone area (cm^2^) in region of interest femur (R1) **(A)**, T9–L5 vertebrae (R2) **(B)** and L1–L6 vertebrae R3 **(C)**; *P* > 0.05, isolated (ISO) versus grouped (GRP) rats (*n* = 10 per group), unpaired student *t*-test**.

Bone mineral density and BMC were evaluated by DEXA in both experimental groups after a period of 4 and 7 weeks of social isolation. Four weeks of social isolation did not induce any alterations (data not shown). Conversely, 7 weeks of social isolation significantly enhanced BMD in R1 (6,5% of increase in BMD for ISO compared to GRP; **Figure 1A**, unpaired *t*-test, *P* = 0.023), R2 (11,3% of increase in BMD for ISO compared to GRP; **Figure 1B**, unpaired *t*-test, *P* = 0.040) and R3 (12,2 % of increase in BMD for ISO compared to GRP; **Figure 1C**, unpaired *t*-test, *P* = 0.025) body scans. However, BMC and bone area were not affected by experimental procedure (**Figures 2** and **3A–C**, unpaired *t*-test, *P* = 0.064, *P* = 0.097, *P* = 0.64 for R1, R2, and R3, respectively). To verify whether the results regarding BMD were a consequence of our study, we performed a regression analyses of BMC versus bone area. After natural logarithm normalization, statistical analyses revealed that those parameters were not proportional (coefficient: 0.21, standard error: 1.47, stat *t*: 1.45, *P*: 0.89).

### Effects of Social Isolation on Bone Thickness

**FIGURE 4:**
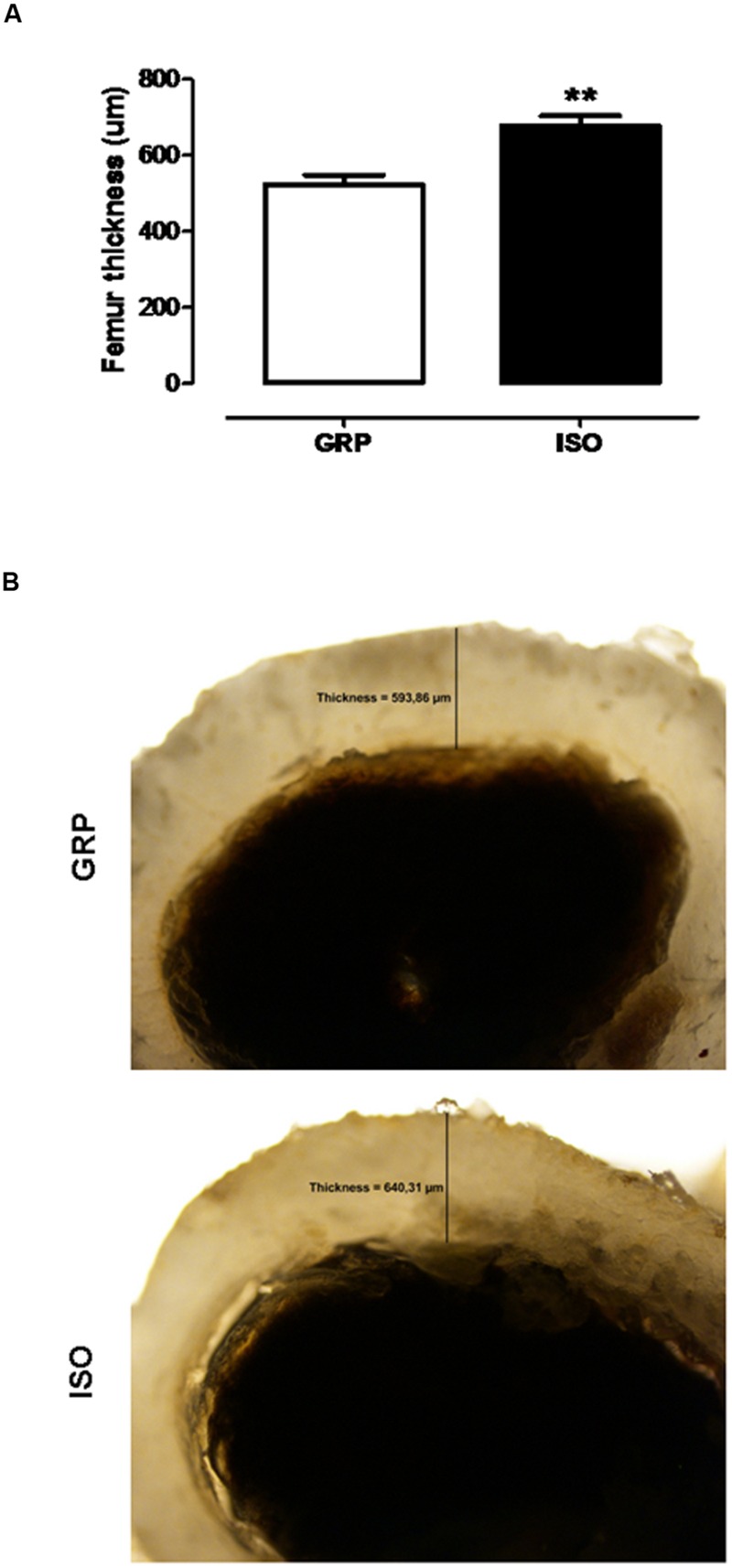
**Effect of social isolation on femur thickness: Effect of 7 weeks of social isolation rearing on rat femur thickness (μm) **(A)**, ^∗∗^*P* < 0.01, isolated (ISO) versus grouped (GRP) rats (*n* = 5–6 per group), unpaired student *t*-test.** DAB-histochemistry of transverse femur section (0.2 cm) of GRP (mean thickness 593.86 μm) and ISO (mean thickness 640.31 μm) rats **(B)**.

To investigate the possible effects of 7 weeks of social isolation on bone thickness, transverse sections from isolated and control rat femora were obtained, colored by DAB-histochemistry, and microscopically analyzed for thickness. A period of 7 weeks of social isolation induced a significant increase of femur thickness compared to control animals (unpaired *t*-test, *P* = 0.003; **Figures 4A,B**), suggesting the development of a hyperostosis condition induced by chronic psychosocial stress.

### Alterations in Bone Formation and Resorption Markers Induced by Social Isolation

To investigate the possible pathological alterations in markers of bone formation and resorption, serum levels of cathepsin K, CTX-I, and sclerostin were determined by ELISA procedure.

**FIGURE 5:**
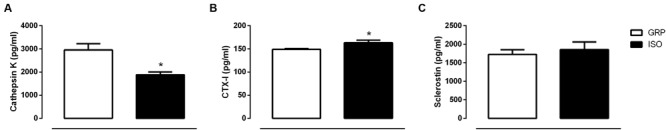
**Effect of social isolation on markers of bone remodeling: Effect of 7 weeks of social isolation rearing on serum cathepsin K levels (pg/ml) **(A)**, cross-linking telopeptide of type I collagen CTX-I (pg/ml) **(B)** and sclerostin (pg/ml) **(C)**.** ^∗^*P* < 0.05, isolated (ISO) versus grouped (GRP) rats (*n* = 5 per group), unpaired student *t*-test.

A period of 4 weeks of social isolation did not alter any of these three markers with respect to control animals (data not shown, unpaired *t*-test, *P* = 0.76, *P* = 0.305, *P* = 0.241 for cathepsin K, CTX-I, and sclerostin, respectively), but a significant decrease of cathepsin K (**Figure 5A**, unpaired *t*-test, *P* = 0.030) as well as a significant increase of CTX-I (**Figure 5B**, unpaired *t*-test, *P* = 0.044) were observed after 7 weeks of social isolation. Sclerostin levels remained stable after the same isolation period compared to controls (**Figure 5C**, unpaired *t*-test, *P* = 0.143).

## Discussion

To the best of our knowledge, this study provides the first *in vivo* evidence for bone status alterations in a rat model of psychosis, the post weaning social isolation. We found that 7 weeks of social isolation lead to increased BMD values either in femoral, trabecular 1 or trabecular 2 sections. This was accompanied by increased cortical femoral thickness, at the same time point. We also identified a significant reduction of serum cathepsin K levels and increased CTX-I serum concentrations after 7 weeks of social isolation, while sclerostin concentrations remained stable. No effects were observed after 4 weeks of isolation rearing.

Bone mineral density in isolated animals increased without corresponding changes either in BMC or bone area. It is known that BMD value can be considered an artificially derived index (Prentice et al., 1994). Therefore, the finding of an increased BMD in isolated animals, without concomitant changes in both BMC and bone area, prompted us to verify whether this observation might have resulted from an artifact. To this purpose, the use of a regression analysis of BMC on bone area, after the conversion of the variables to natural logarithms (Prentice et al., 1990; Van Loan et al., 1998), was necessary to obtain a regression coefficient, providing, finally, the appropriate power coefficient. Thus, by determining the power relation between BMC and bone area, we assessed the degree whereby BMD increase did not match a concomitant increase in BMC and bone area. After BMC and bone area conversion to natural logarithms, BMC was regressed against bone area. The obtained regression coefficient value (0,21) supported the fact that, under our experimental conditions, BMC and bone area were not directly proportional to each other. These results appeared to be in line with previous reports showing that BMC is an independent variable, not directly proportional to bone area. Consequently, a variation in BMC or bone area is not reflected in a corresponding and concomitant alteration in BMD, meaning that changes in BMC and bone area can be observed independently of each other (Svendsen et al., 1993; Prentice et al., 1994; Van Loan et al., 1998). Further on, our findings were also supported by our results on sclerostin levels. Thus, the lack of alterations in serum sclerostin concentrations were in favor of no observed alterations either in BMC and bone area. Indeed, sclerostin is known to be predominately expressed by osteocytes, displaying anti-anabolic effects on bone formation (Gooi et al., 2010; Atkins et al., 2011; Kogawa et al., 2013; Compton and Lee, 2014). In this regard, sclerostin knockout mice have been demonstrated to exhibit an increase in bone mass (Lewiecki, 2014; Ryan et al., 2015).

Rat hyperactivity in a novel environment has been used as a translational feature of psychotic agitation (Miyakawa et al., 2003; Powell and Miyakawa, 2006; Porsolt et al., 2010; Jones et al., 2011). Accordingly, we have previously demonstrated that social isolation in rodents is associated to increased spontaneous locomotor activity (Schiavone et al., 2012; Colaianna et al., 2013). Such hyperactivity could account for the observed increase of BMD. Further on, our results are in line with previous studies emphasizing the importance of physical activity for bone health (Bourrin et al., 1995; Iwamoto et al., 2005; Morgan and Weiss Jarrett, 2011; Heidemann et al., 2015; Ryan and Shaw, 2015). On the other hand, sedentary behavior and physical inactivity may change the body composition, including BMD (Heidemann et al., 2013). Thus, gain in BMD might respond to physical hyperactivity in terms of increased mechanical loading in isolated rats.

After performing a microscopy evaluation of bone thickness in control and isolated rats, we observed a significant increase of femoral cortex thickness after 7 weeks of isolation rearing, suggesting that social isolation significantly affects bone formation, inducing a hyperostosis-like pattern.

The mechanism by which social isolation induced a hyperostosis condition is not yet clear, but the present results raise the possibility that an imbalance between osteoclastic bone resorption and osteoblastic bone formation is involved. Indeed, to assess directly whether isolation rearing affects bone remodeling, as the above data suggest, we measured serum markers of bone turnover, such as cathepsin K and CTX-I. As expected, our data showed that isolation rearing induced a significant decrease of serum cathepsin K levels. Normal bone resorption critically depends on the synthesis and secretion of cathepsin K, a protease, predominantly expressed in osteoclasts, with a prominent role in bone remodeling (Troen, 2004; Yasuda et al., 2005; Brix et al., 2008). Osteoclasts isolated from cathepsin K knockout mice exhibit impaired bone resorption *in vitro* (Troen, 2004). On the other hand, cathepsin K hyperactivity has been linked to osteoporosis (Kiviranta et al., 2001). Thus, in our experimental conditions, we hypothesize that the influence of social isolation on BMD could, at least in part, be mediated by a reduction in cathepsin K levels, given that bone resorption depends upon the synthesis of cathepsin K by osteoclasts. Intriguingly, an important role of cathepsin K deficiency on learning and memory processes, as well as novelty seeking behavior, has been recently identified. In particular, cathepsin K deficient mice exhibited marked memory impairments in behavioral assessments, as indicated by their inability to discriminate the introduction of a novel object into a familiar environment (Dauth et al., 2011). Likewise, we have previously demonstrated that, while control rats were able to recognize the novel object from the familiar one over the 7-week period, the exploratory activity of isolated animals was compromised (Schiavone et al., 2012; Colaianna et al., 2013). Moreover, analysis of *postmortem* brain samples from schizophrenic patients has shown up-regulation of cathepsin K (Bernstein et al., 2007). However, the observed increased expression of cathepsin K might likely be the effect of the long-term medication, rather than a result of the underlying disease. Unfortunately, no data on the expression of cathepsin K in drug-naive psychotic patients exist. Thus, in the light of the above findings, we propose that cathepsin K activity disruption, during development and adulthood, exerts a substantial impact on the functional integrity of the rat brain, eventually resulting in learning and memory deficits, as well as in bone homeostasis impairments. In this regard, we suggest that social isolation could provide a useful tool to better understand the role that cathepsin K seems to play in the brain, in addition to its function in the turnover of bone tissue.

Degradation products derived from osteoclastic resorption of the bone matrix could be used as a sensitive index of the resorption process. High specificity as a bone resorption marker is provided by a biochemical assay for degradation fragments of the CTX-I (Okabe et al., 2001).

Indeed, in our study, this represented another important factor that underwent significant increase in isolated animals. Despite an increase in CTX-I levels, the end effect observed in our experimental conditions was an increase in BMD and bone thickness. With respect to this aspect, it should be taken into account that CTX-I is eliminated by kidney filtration. In this context, it has been recently demonstrated that early life stress, such as maternal separation and social isolation, is associated to renal dysfunctions in rats (Loria et al., 2013). Importantly, it has also been reported that an increase of oxidative stress in rodents could lead to chronic renal dysfunctions (Aragno et al., 2003; Atessahin et al., 2007; Shah and Iqbal, 2010). Accordingly, we previously demonstrated the presence of increased oxidative stress in isolated animals (Schiavone et al., 2009). Thereby, it could be hypothesized that, in isolated animals, the increase in CTX-I levels does not depend directly on bone resorption as a primary phenomenon. Rather, it may arise from a possible isolation-induced renal dysfunction and decreased urinary excretion. Further, bone resorption, associated to CTX-I increase, might be biologically balanced by bone formation induced by hyperlocomotion (Falcai et al., 2015; Sioen et al., 2015), which is a specific behavioral trait of isolated animals, as previously reported by our group and others (Fone and Porkess, 2008; Levine et al., 2008; Schiavone et al., 2009, 2012). The interaction and the counterbalance between these two aspects (possible renal failure and hyperlocomotion) might explain, at least in part, why, despite an increase in CTX-I levels, isolated rats showed increased bone thickness and BMD.

In the context of possible cathepsin K, CTX-I and sclerostin interactions, several pathophysiological pathways should be considered. Among them, we have previously reported a crucial role of the NADPH oxidase NOX2-derived central and peripheral oxidative stress in the pathogenesis of neuropathological alterations induced by social isolation (Schiavone et al., 2009, 2012, 2015). Thus, we could not exclude that increased oxidative stress in isolated rats might contribute to alterations of bone homeostasis. This hypothesis is also supported by previous investigations, showing that osteoclastogenesis is stimulated, mediated and regulated by reactive oxygen species (Kondo et al., 2013; Callaway and Jiang, 2015). Importantly, a previous study reported a crucial role of NOX2 enzyme in bone resorption, thus osteoclasts from NOX2 knock-out mice produced the same amount of superoxide and do not exhibit signs of osteopetrosis (Yang et al., 2001). Other NOX enzymes, such as NOX1 and NOX4 have also been shown to play a role in regulation of bone homeostasis (Yang et al., 2004; Sasaki et al., 2009). Mitochondrial-derived oxidative stress (Guha et al., 2016) as well as inflammatory pathways (Pislar and Kos, 2014) leading to pathological modifications of dopaminergic function should also be considered. Furthermore, it should be taken into account that the primary outcomes of the social isolation are changes in the levels of stress mediators. Importantly, many of these stress mediators have profound effects on bone homeostasis, including regulation of osteoclast differentiation and activity. These include mediators of hypothalamic pituitary-adrenal (HPA) axis response (mainly cortisol in humans and corticosterone in rodents) and sympathetic neurotransmitters (catecholamines, neuropeptide Y). In this regard, we previously showed that psychosocial stress determines alterations of the HPA-axis functioning (Colaianna et al., 2013). In particular, elevations in the hypothalamic levels of corticotropin-releasing factor and plasmatic adrenocorticotropic hormone were observed from 4 weeks of social isolation, and increased levels of plasmatic and salivary corticosterone were found at a later time point of social isolation (7 weeks). We also showed that chronic stress resulted in altered monoamine levels that vary according to brain area and rat strain (Trabace et al., 2012). Another crucial stress mediator, whose increase has been shown in the social isolation model (Thorsell et al., 2006), is the neuropeptide Y. Thus a central role of this peptide in the coordination of bone mass and weight, in the control of osteoblast function and regulation of bone mass as well as in bone remodeling under chronic stress conditions has been demonstrated (Elefteriou, 2008; Baldock et al., 2009).

To our knowledge, there are no published reports on skeletal status related to chronic social stress-induced psychosis. Among several comorbidities associated with psychosis, bone health has only recently received attention. Indeed, only few studies have focused on bone homeostasis in psychosis and suggest that the pathology seems to be associated with low BMD in humans (Meyer and Lehman, 2006). In a Danish case-control study, antipsychotic treatment was associated with an increase in overall risk of fracture (Vestergaard et al., 2006). Similarly, Renn et al. (2009) showed that schizophrenic patients had lower bone mass than young community population, in terms of bone density measured as broadband ultrasound attenuation. Moreover, an increased risk of osteoporosis and fragility fractures in medicated psychotic patients has been reported (Partti et al., 2010; Graham et al., 2011). Significantly lower calcaneal ultrasound values were also observed in subjects treated with antipsychotic or mood-stabilizing compounds (Partti et al., 2010). In the same line, clozapine, a common antipsychotic drug, exerts adverse skeletal effects in rodents (Costa et al., 2011).

All available data are referred to medicated patients and the observed effects on bone homeostasis are usually considered to be an adverse effect of antipsychotic therapy. Interestingly, our findings suggest that, at least in part, the skeletal status abnormalities could represent a pre-existing condition. Accordingly, Kaifu et al. (2003) demonstrated, in an elegant work, that DAP12^-/-^ mice, characterized by a combination of bone alterations and psychotic symptoms, developed an increased bone mass, in which bone resorption was impaired because of decreased osteoclastic activity. On the other hand, the mutant mice also exhibited a reduced startle response, as well as an impaired pre-pulse inhibition to acoustic stimuli, indicating the deficits commonly observed in several neuropsychiatric diseases, such as schizophrenia in humans. In this context, our data could have important implications since they may contribute to recognize that, in antipsychotic-naive subjects, possible skeletal status alterations might be caused by high levels of chronic psychological stress. The animal model paradigm used in our study permitted to exclude all factors that undoubtedly contribute to alter BMD in psychotic patients, firstly therapy, but also unhealthy lifestyle behaviors, including unhealthy diet, alcoholism or smoking.

## Conclusion

Our observations suggest that chronic psychosocial stress might impair bone integrity. Thus, before the beginning of a new anti-psychotic treatment, several patient-related clinical aspects should be taken into account, such as the need to control the coexistence of obesity and hypertension. Moreover, bone health status of psychotic patients should be deeply evaluated before the introduction of a new antipsychotic therapy. To this purpose, the social isolation model might represent a useful tool to better investigate molecular mechanisms leading to hyperostosis and to possibly define new therapeutic strategies.

## Author Contributions

Study design: LT and VC. Study conduct: SS, MM, EM, MB, and PT. Data collection: SS, MM, EM, MB, and PT. Data analysis: SS, MM, EM, MB, and PT. Data interpretation: SS, MM, EM, PT, ADG, FC, and NM. Drafting manuscript: SS, MM, EM, and LT. Revising manuscript content: SS, MM, EM, MB, CC, PT, ADG, FC, NM, LT, and VC. Approving final version of the manuscript: SS, MM, EM, MB, CC, PT, ADG, FC, NM, LT, and VC. LT takes responsibility for the integrity of the data analysis.

## Conflict of Interest Statement

The authors declare that the research was conducted in the absence of any commercial or financial relationships that could be construed as a potential conflict of interest.
